# 28-Day Oral Toxicity of Cadmium Selenide in Sprague-Dawley Rats

**DOI:** 10.5487/TR.2009.25.3.140

**Published:** 2009-09-01

**Authors:** Yong Soon Kim, Moon Yong Song, Jin Sik Kim, Dae Sik Rha, Yong Joon Jeon, Ji Eun Kim, Hyeon Yeol Ryu, Il Je Yu, Kyung Seuk Song

**Affiliations:** 15 Department of Toxicopathology, Korea Environment & Merchandise Testing Institute, Songdo-dong 7–44, Yeounsu-gu, Incheon, 406-840 Korea; 25 Fusion Technology Research Institute, Hoseo University, Chungnam, 336-795 Korea

**Keywords:** 28-Oral administration, Toxicology, Cadmium selenide, Rat, ISO 10993-11

## Abstract

This study was performed to evaluate the toxicity of cadmium selenide for a period of 28 days in Sprague-Dawley rats. Each of 10 healthy male and females rats per group received daily oral administration for 28-day period at dosage levels 30, 300 and 1,000 mg/kg of body weight. Mortality and clinical signs were checked, and body weight, water intake and food consumption were also recorded weekly. There were no dose-related changes in food consumption or urine volume. All animals survived to the end of study with no clinical signs or differences in body weight gain observed when compared with the control group. At the end of study, all animals including control group, were subjected to necropsy. Blood samples were collected for hematology tests including coagulation time and biochemistry analysis. Blood coagulation time and relative organ weight were unaffected by all received doses. White Blood Cell (WBC) counts significantly increased in the 300 mg/ kg administered male animal group when compared to the control. Monocyte (MO) value were also increased significantly in both 300 and 1,000 mg/kg male animal group. However, Mean Corpuscular Volume (MCV) were significantly decreased compared with the control in the 1,000 mg/kg dose groups for male and female animals. Mean Corpuscular Hemoglobin (MCH) decreased significantly for female in the 300 and 1,000 mg/kg group compared to the control. Blood biochemical values of Inorganic phosphorus (IP) were significantly increased in both the 300 and 1,000 mg/kg dose groups in male animals when compared to the control. Creatinine (CRE) levels indicated significant increase in kidney function for the female, 30 mg/kg dose group when compared with control. There was a significant decrease in thymus absolute organ weight in the female, 1,000 mg/kg dose group when compared with control. Histopathological findings revealed no evidence of injury related to cadmium selenide except for one case of focal hepatic inflammation in the high dose (1,000 mg/kg) group. One case of lung inflammation was also seen in the control group. Basis on these result, the No Observable Adverse Effect Level (NOAEL) of cadmium selenide was determined to be more than 1,000 mg/kg/day for male and female rats under conditions in this study.

## INTRODUCTION

Nano-technology is an applied science, a rapidly growing industry generating a diverse array of nano-scale materials and process (Ron Hardman, 2006; Tang *et al*., 2008; Donaldson *et al*., 2006). Quantum dots are one of well known nano-scale materials with wide-spread application in field such as medicine, plastics, energy *etc*. Quantum dots are semiconductor nano-crystals with unique optical and electrical properties currently applied in biomedical imaging and electronics industries (Bruchez *et al*., 1998). Previous studies showed some toxicity information about quantum dots (Ron Hardman, 2006; Tang *et al*., 2008). However, quantum dots toxicity is controversial because of the diversity quantum dots being synthesized and physico-chemical characteristics are referred to as core-shell-conjugate such as cadmium selenide, zinc sulfide *etc*. Cadmium selenide (CdSe) is one of composed material for core shell of quantum dots. CdSe is the promising semiconductor material for its wide range of technological applications in optoelectronics devices (Kale *et al*., 2005; Lokhande *et al*., 2005). In this study, we performed to evaluate the toxicity of CdSe, one of composition material for quantum dots. Sprague-Dawley (SD) rats were administrated to CdSe for a period of 28 days. This study was conducted according to the test guidelines from ISO 10993 "Biological evaluation of medical devices-part 11: Test for systemic toxicity" (International standard, 2006).

## MATERIALS AND METHODS

***Test substance.*** The Cadmium selenide (average particle size: 5.60 μm) were purchased from Sigma-Aldrich (USA) and were at least 99.99% pure. 0.5% aqueous carboxy methylcellulose (CMC) was also obtained from SIGMA-ALDRICH (USA) for the vehicle.

***Animals and environmental conditions.*** Four-weeks-old male and female, specific pathogen-free (SPF) Sprague-Dawley (SD) rats were purchased from Orient Bio Inc. (Korea) and acclimated for 8 days for male, 9 days for female before starting the experiments. During the acclimation and experimental periods, the rats were housed in polycarbonate cages (2 rats per cage) in an animal room with controlled temperature (22 ± 3°C) and humidity (50 ± 20%), and a 12-hr light/dark cycle. The rats were fed rodent chow (Harlan Teklad, USA) and filtered water *ad libitum*. Acclimation and all animal experimental procedure were approved by the Institute Animal Care and Use Committee of KEMTI.

***Experimental design.*** After acclimimation, the rats of each sex were divided into four groups (10 rats in each group): vehicle control (0.5% carboxy methyl cellulose), low dose (30 mg/kg/day), middle dose (300 mg/kg/day), and high dose group (1,000 mg/kg/day). The animals were orally administered cadmium selenide following ISO 10993 "Biological evaluation of medical devices-part 11: Test for systemic toxicity" (International standard, 2006) based on repeated exposure systemic toxicity.

***Ophthalmoscopy.*** All animals were subjected to ophthalmologic examination before the start of the administration and the end of study with slit lamp (RC-2, Kowa. ltd., Japan).

***Urinalysis.*** Overnight urine samples were collected from all animals under food and water deprivation once at the end of the administration and analyzed for bilirubin, blood pigments, glucose, ketones, leukocytes, nitrites, pH, protein and urobilinogen. In addition, volume, color, appearance and specific gravity were recorded with Multistix 10SG (SIEMENS, German).

***Biochemistry and hematology.*** Before necropsy, food was withheld for overnight. And the rats were anesthetized by $CO_2$ gas inhalation after recording the terminal body weights. Blood samples were drawn from the descending aorta, collected in heparinized vacutainers, and then analyzed for ALB (albumin), ALP (alkaline phosphatase), Ca (calcium), CHO (cholesterol), CRE (creatinine), gamma-GT (gamma-glutamyl transpeptidase), GLU (glucose), AST (aspartate aminotransferase), ALT (alanine aminotransferase), LDH (lactate dehydrogenase), MG (magnesium), TP (total protein), UA (uric acid), BUN (blood urea nitrogen), T-BIL(total bilirubin), IP (inorganic phosphorus), TG (triglyceride), CPK (creatine phosphokinase), Na (sodium), K (potassium) and Cl (chloride) using a biochemical blood analyzer (Hitachi 1780, Hitachi, Japan). The blood was also analyzed for the WBC (white blood cell count), RBC (red blood cell count), Hb (hemoglobin concentration), HTC (hematocrit), MCV (mean corpuscular volume), MCH (mean corpuscular hemoglobin), MCHC (mean corpuscular hemoglobin concentration), RDW (red cell distribution width), PLT (platelet counts), MPV (mean platelet volume), NE % (percent of neutrophils), LY % (percent of lymphocytes), MO % (percent of monocytes), EO % (percent of eosinophils) and BASO % (percent of basophils) using a blood cell counter (Hemavet 0950, CDC Tech., USA).

***Organ weights and histopathology.*** After collecting the blood, all animals were conducted to euthanasia by $CO_2$ gas at the end of the study. The adrenal glands, testes, ovaries, uterus, heart, thymus, prostate, lungs, kidneys, spleen, liver, pituitary gland and brain were all removed carefully. These organs were then weighed and fixed in a 10% Neutral bufferd formalin. Thereafter, the organs were embedded in paraffin and cut. The sections were stained with hematoxylin and eosin, and examined under light microscopy.

***Statistical analysis.*** The differences between the groups were examined using the standard one-way analysis of variance (ANOVA). If these test showed statistical significance, the data was analyzed using the multiple comparison procedure Duncan's or Dunnett's multiple range test were used to compare the body weights, organ weights, and results of the blood biochemistry and hematology for the three experimental groups with those for control. SPSS for Windows 12.0 K

software package was used. A value of $p < 0.05$ indicated statistical significance.

## RESULTS

***Clinical signs, food and water consumption.*** No mortality and notable clinical signs were observed during the 28-day exposure period and there were no significant differences in food and water consumption between the administered and control group (data not shown).

***Body weight changes.*** There was no statistically significant change in body weight of administered animals compared to the control group (Fig. 1 and Fig. 2).

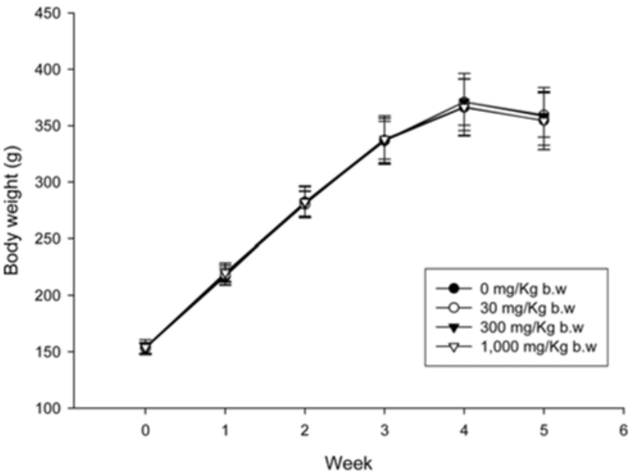

**Fig. 1.** Body weight changes of male rats in the 28 days oral administration with cadmium selenide. Four groups of 10 male rats were exposed to cadmium selenide at concentrations of 0, 30, 300 and 1,000 mg/kg/day, 7 days/week for 28 days. The rats exposed to the cadmium selenide showed no significant differences as compared with the control.

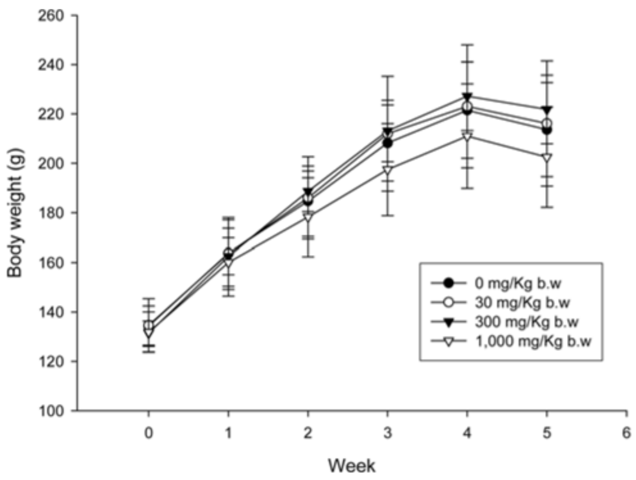

**Fig. 2.** Body weight changes of female rats in the 28 days oral administration with cadmium selenide. Four groups of 10 female rats were exposed to cadmium selenide at concentrations of 0, 30, 300 and 1,000 mg/kg/day, 7 days/week for 28 days. The rats exposed to the cadmium selenide showed no significant differences as compared with the control.

**Table 1.** Hematological data for male rat in the 28 days oral administration with cadmium selenide (Mean ± SD)

| | Dose (mg/Kg) | | | |
|---|---|---|---|---|
| | 0 (n = 9) | 30 (n = 10) | 300 (n = 10) | 1,000 (n = 9) |
| WBC | 12.00 ± 4.35 | 13.35 ± 2.49 | 16.06 ± 2.41* | 13.32 ± 2.82 |
| RBC | 6.93 ± 1.40 | 7.25 ± 0.44 | 7.47 ± 0.40 | 7.39 ± 0.35 |
| Hb | 14.69 ± 3.46 | 15.40 ± 1.04 | 15.97 ± 1.20 | 14.86 ± 1.31 |
| HCT | 35.32 ± 7.39 | 36.29 ± 3.61 | 37.89 ± 3.25 | 34.51 ± 2.89 |
| MCV | 50.97 ± 1.95 | 49.99 ± 3.13 | 50.74 ± 3.37 | 46.71 ± 3.62* |
| MCH | 21.00 ± 2.24 | 21.28 ± 1.55 | 21.42 ± 1.62 | 20.13 ± 1.89 |
| MCHC | 41.36 ± 5.62 | 42.84 ± 5.27 | 42.54 ± 5.86 | 43.42 ± 6.21 |
| RDW | 17.71 ± 1.13 | 18.27 ± 0.95 | 18.11 ± 0.91 | 21.13 ± 2.02** |
| PLT | 938.50 ± 316.94 | 999.30 ± 68.31 | 991.40 ± 128.90 | 1055.67 ± 227.03 |
| MPV | 5.47 ± 0.86 | 5.76 ± 0.39 | 5.51 ± 0.21 | 5.64 ± 0.56 |
| NEU | 5.58 ± 4.94 | 7.96 ± 5.94 | 7.83 ± 7.14 | 7.00 ± 5.09 |
| LYO | 91.51 ± 5.38 | 88.43 ± 6.56 | 88.23 ± 8.24 | 88.88 ± 6.09 |
| MONO | 2.75 ± 0.58 | 3.34 ± 1.08 | 3.78 ± 1.32 | 3.96 ± 1.08 |
| EOS | 0.12 ± 0.14 | 0.21 ± 0.51 | 0.14 ± 0.11 | 0.12 ± 0.11 |
| BASO | 0.04 ± 0.07 | 0.05 ± 0.14 | v0.02 ± 0.03 | 0.03 ± 0.04 |

*Note.* WBC (K/μl), White blood cells; RBC (M/μl), Red blood cells; Hb (g/dl), Hemoglobin; HCT (%), Hematocrits; MCV (fl), Mean corpuscular volume; MCH (pg), Mean corpuscular hemoglobin; MCHC (g/dl), Mean corpuscular hemoglobin concentration; RDW (%), Red cell distribution width; PLT (K/μl), Platelets; MPV (fl), Mean platelet volume; NEU (%), Neutrophils; LYO (%), Lymphocytes; MONO (%), Monocytes; EOS (%), Eosinophils; BASO (%), Basophils.
*Significant difference vs. control, $p < 0.05$.
**Significant difference vs. control, $p < 0.01$.

**Table 2.** Hematological data for female rats in the 28 days oral administration with cadmium selenide (Mean ± SD)

| | Dose (mg/Kg) | | | |
|---|---|---|---|---|
| | 0 (n = 9) | 30 (n = 10) | 300 (n = 10) | 1,000 (n = 9) |
| WBC | 9.54 ± 1.84 | 9.16 ± 2.40 | 9.94 ± 2.71 | 9.81 ± 3.13 |
| RBC | 7.39 ± 0.47 | 7.26 ± 0.25 | 7.58 ± 0.45 | 7.73 ± 0.42 |
| Hb | 16.51 ± 1.02 | 16.12 ± 0.53 | 16.36 ± 0.80 | 16.62 ± 0.99 |
| HCT | 32.60 ± 1.78 | 31.84 ± 0.86 | 32.78 ± 1.93 | 32.60 ± 2.13 |
| MCV | 44.18 ± 1.38 | 43.84 ± 1.13 | 43.26 ± 1.04 | 42.14 ± 1.53** |
| MCH | 22.36 ± 0.66 | 22.22 ± 0.74 | 21.59 ± 0.54* | 21.49 ± 0.85* |
| MCHC | 50.62 ± 0.93 | 50.63 ± 0.89 | 49.93 ± 0.64 | 51.02 ± 1.17 |
| RDW | 16.02 ± 1.07 | 16.02 ± 0.80 | 15.44 ± 0.56 | 16.51 ± 0.91 |
| PLT | 910.67 ± 202.89 | 992.60 ± 64.27 | 940.33 ± 100.99 | 1004.78 ± 100.81 |
| MPV | 5.93 ± 0.63 | 5.98 ± 0.31 | 5.92 ± 0.25 | 5.88 ± 0.27 |
| NEU | 8.93 ± 3.53 | 8.02 ± 2.78 | 7.02 ± 3.10 | 9.16 ± 4.55 |
| LYO | 87.12 ± 4.05 | 88.38 ± 2.95 | 89.10 ± 4.04 | 87.21 ± 5.06 |
| MONO | 3.67 ± 0.72 | 3.43 ± 0.72 | 3.76 ± 1.22 | 3.48 ± 1.09 |
| EOS | 0.16 ± 0.22 | 0.13 ± 0.10 | 0.10 ± 0.12 | 0.12 ± 0.14 |
| BASO | 0.11 ± 0.16 | 0.05 ± 0.06 | 0.02 ± 0.02 | 0.04 ± 0.04 |

*Note.* WBC (K/μl), White blood cells; RBC (M/μl), Red blood cells; Hb (g/dl), Hemoglobin; HCT (%), Hematocrits; MCV (fl), Mean corpuscular volume; MCH (pg), Mean corpuscular hemoglobin; MCHC (g/dl), Mean corpuscular hemoglobin concentration; RDW (%), Red cell distribution width; PLT (K/μl), Platelets; MPV (fl), Mean platelet volume; NEU (%), Neutrophils; LYO (%), Lymphocytes; MONO (%), Monocytes; EOS (%), Eosinophils; BASO (%), Basophils.
*Significant difference vs. control, $p < 0.05$.
**Significant difference vs. control, $p < 0.01$.

**Table 3.** Blood biochemistry data for male rats in the 28 days oral administration with cadmium selenide (Mean ± SD)

| | Dose (mg/Kg) | | | |
|---|---|---|---|---|
| | 0 (n = 10) | 30 (n = 10) | 300 (n = 10) | 1,000 (n = 9) |
| ALB | 2.81 ± 0.13 | 2.73 ± 0.08 | 2.77 ± 0.21 | 2.90 ± 0.25 |
| ALP | 916.50 ± 187.38 | 975.70 ± 87.48 | 901.60 ± 148.62 | 862.00 ± 152.15 |
| CA | 12.93 ± 0.46 | 12.87 ± 0.47 | 12.90 ± 0.45 | 12.98 ± 0.49 |
| CHO | 100.20 ± 18.97 | 95.80 ± 21.49 | 89.89 ± 13.78 | 99.22 ± 23.67 |
| CRE | 0.78 ± 0.06 | 0.79 ± 0.17 | 0.75 ± 0.11 | 0.86 ± 0.05 |
| GGT | 0.00 ± 0.00 | 0.20 ± 0.42 | 0.00 ± 0.00 | 0.22 ± 0.44 |
| GLU | 162.70 ± 35.88 | 171.60 ± 16.06 | 146.90 ± 30.76 | 169.00 ± 20.40 |
| AST | 102.60 ± 20.80 | 147.90 ± 98.99 | 113.70 ± 17.33 | 117.11 ± 12.40 |
| ALT | 47.90 ± 8.60 | 69.10 ± 48.56 | 58.00 ± 9.36 | 50.78 ± 7.69 |
| LDH | 521.40 ± 379.72 | 742.40 ± 513.31 | 614.00 ± 290.83 | 744.22 ± 379.79 |
| MG | 3.66 ± 0.34 | 3.73 ± 0.37 | 4.10 ± 0.42 | 3.63 ± 1.03 |
| TP | 6.82 ± 0.28 | 6.81 ± 0.38 | 6.89 ± 0.24 | 6.94 ± 0.32 |
| UA | 3.18 ± 0.78 | 3.12 ± 0.68 | 3.86 ± 0.80 | 3.71 ± 0.95 |
| BUN | 20.48 ± 4.66 | 20.34 ± 2.67 | 19.13 ± 4.78 | 19.09 ± 2.68 |
| T-BIL | 0.02 ± 0.02 | 0.01 ± 0.02 | 0.01 ± 0.01 | 0.02 ± 0.02 |
| IP | 12.95 ± 1.03 | 13.33 ± 0.86 | 14.38 ± 1.43* | 14.27 ± 1.46* |
| TG | 46.40 ± 23.77 | 34.60 ± 7.55 | 36.50 ± 14.92 | 51.89 ± 46.29 |
| CPK | 435.20 ± 223.22 | 560.20 ± 312.93 | 441.20 ± 148.53 | 542.78 ± 291.95 |
| Na | 159.20 ± 1.75 | 159.20 ± 1.69 | 158.60 ± 2.01 | 158.67 ± 2.12 |
| K | 5.77 ± 1.20 | 5.97 ± 0.86 | 6.53 ± 1.67 | 6.62 ± 1.57 |
| Cl | 113.30 ± 0.82 | 113.00 ± 1.15 | 113.00 ± 1.49 | 112.56 ± 0.88 |

*Note.* ALB (g/dl), Albumin; ALP (IU/l), Alkaline phosphatase; CA (mg/dl), Calcium; CHO (mg/dl), Total cholesterol; CRE (mg/dl), Creatinine; GGT (IU/l), Gamma glutamyl transpeptidase; GLU (mg/dl), Glucose; AST (IU/l), Aspartate aminotransferase; ALT (IU/l), Alanine aminotransferase; LDH (IU/l), Lactate dehydrogenase; MG (mg/dl), Magnesium; TP (g/dl), Total protein; UA (mg/dl), Uric acid; BUN (mg/dl), Blood urea nitrogen; T-BIL (mg/dl), Total bilirubin; IP (mg/dl), Inorganic phosphorus; TG (mg/dl), Triglyceride; CPK (U/l), Creatine phosphokinase; Na (mmol/l), Sodium; K (mmol/l), Potassium; Cl (mmol/l), Chloride.
*Significant difference vs. control. $p < 0.05$.

***Ophthalmoscopy.*** Ophthalmologic examination did not reveal in any of the animals (data not shown).

***Urinalysis.*** Statistically significant increase ($p < 0.05$) in frequency of leukocyte was noted in male 300 and 1,000 mg/kg group compared with control. And significant increase ($p < 0.05$) in frequency of protein was noted in male 1,000 mg/kg group compared to the control. But, there were no statistically significant changes in female rats. No significant changes in urine volume and sediment examination were observed (data not shown).

***Effects on hematology.*** In male rats, significant increase in white blood cell (WBC, $p < 0.05$) and monocyte (MO, $p < 0.05$) values in 300 mg/kg group, and MO ($p < 0.05$) and red cell distribution width (RDW, $p < 0.01$) in 1,000 mg/kg group were noted compared to the control. But, significant decrease in mean corpuscular volume (MCV, $p < 0.05$) in 1,000 mg/kg group was noted compared to the control (Table 1). In female rats, there were significant decrease in mean corpuscular hemoglobin (MCH, $p < 0.05$) in 300 mg/kg group, MCV ($p < 0.01$) and MCH ($p < 0.05$) were significantly decreased in 1,000 mg/kg group compare to the control (Table 2).

***Coagulation time.*** No significant difference between administered and control group was seen for prothrombin time (PT) and active partial thromboplastin time (APTT) (data not shown).

***Effects on blood biochemistry.*** In male rats, there were significant increase in inorganic phosphorus (IP, $p < 0.05$) in both 300 and 1,000 mg/kg group (Table 3). In female rats, there was increase in creatinine (CRE, $p < 0.05$) in 30 mg/kg group (Table 4).

***Gross findings.*** At necropsy, there were no prominent changes in any treated groups of both sexes (data not shown).

***Organ weights.*** Decreased absolute weight of thymus in female 1,000 mg/kg group was observed compared to the control (data not shown).

***Histopathological examination.*** There was one focal inflammation in liver for male 1,000 mg/kg group, and there was on focal inflammation in lung for male

**Table 4.** Blood biochemistry data for female rats in the 28 days oral administration with cadmium selenide (Mean ± SD)

| | Dose (mg/Kg) | | | |
|---|---|---|---|---|
| | 0 (n = 10) | 30 (n = 10) | 300 (n = 10) | 1,000 (n = 10) |
| ALB | 2.96 ± 0.23 | 3.05 ± 0.16 | 3.00 ± 0.19 | 3.09 ± 0.22 |
| ALP | 543.00 ± 108.75 | 532.40 ± 71.11 | 535.30 ± 103.40 | 532.70 ± 115.99 |
| CA | 18.78 ± 0.77 | 18.81 ± 1.37 | 18.60 ± 1.19 | 18.97 ± 1.70 |
| CHO | 122.30 ± 21.08 | 108.30 ± 22.33 | 121.80 ± 23.66 | 102.70 ± 18.54 |
| CRE | 0.79 ± 0.11 | 0.94 ± 0.12* | 0.86 ± 0.11 | 0.88 ± 0.09 |
| GGT | 0.30 ± 0.48 | 0.20 ± 0.42 | 0.40 ± 0.52 | 0.70 ± 0.48 |
| GLU | 146.30 ± 47.11 | 130.70 ± 26.82 | 124.30 ± 27.76 | 123.80 ± 27.90 |
| AST | 119.90 ± 24.39 | 136.00 ± 34.01 | 127.60 ± 35.69 | 118.70 ± 35.83 |
| ALT | 40.50 ± 8.50 | 42.40 ± 6.40 | 46.70 ± 13.28 | 38.20 ± 6.68 |
| LDH | 952.70 ± 480.93 | 1197.30 ± 692.66 | 937.40 ± 797.74 | 804.90 ± 753.63 |
| MG | 4.00 ± 0.30 | 4.08 ± 0.53 | 4.00 ± 0.42 | 4.19 ± 0.35 |
| TP | 6.92 ± 0.34 | 7.13 ± 0.32 | 7.00 ± 0.41 | 7.21 ± 0.38 |
| UA | 2.71 ± 0.61 | 2.84 ± 0.82 | 2.95 ± 0.36 | 2.84 ± 0.40 |
| BUN | 21.85 ± 2.42 | 22.03 ± 4.47 | 23.83 ± 5.17 | 22.18 ± 4.34 |
| T-BIL | 0.02 ± 0.02 | 0.03 ± 0.01 | 0.03 ± 0.02 | 0.02 ± 0.02 |
| IP | 13.40 ± 1.26 | 13.70 ± 1.50 | 13.07 ± 1.68 | 13.15 ± 1.20 |
| TG | 24.40 ± 11.53 | 15.60 ± 13.06 | 15.90 ± 6.72 | 12.50 ± 8.73 |
| CPK | 577.40 ± 234.34 | 715.60 ± 341.50 | 559.50 ± 382.14 | 496.70 ± 383.63 |
| Na | 159.00 ± 1.33 | 159.00 ± 1.15 | 158.70 ± 0.95 | 159.20 ± 2.90 |
| K | 4.93 ± 0.68 | 5.13 ± 0.83 | 4.88 ± 0.41 | 4.93 ± 0.42 |
| Cl | 113.90 ± 1.37 | 114.40 ± 1.71 | 114.00 ± 1.41 | 114.0 ± 2.16 |

*Note.* ALB (g/dl), Albumin; ALP (IU/l), Alkaline phosphatase; CA (mg/dl), Calcium; CHO (mg/dl), Total cholesterol; CRE (mg/dl), Creatinine; GGT (IU/l), Gamma glutamyl transpeptidase; GLU (mg/dl), Glucose; AST (IU/l), Aspartate aminotransferase; ALT (IU/l), Alanine aminotransferase; LDH (IU/l), Lactate dehydrogenase; MG (mg/dl), Magnesium; TP (g/dl), Total protein; UA (mg/dl), Uric acid; BUN (mg/dl), Blood urea nitrogen; T-BIL (mg/dl), Total bilirubin; IP (mg/dl), Inorganic phosphorus; TG (mg/dl), Triglyceride; CPK (U/l), Creatine phosphokinase; Na (mmol/l), Sodium; K (mmol/l), Potassium; Cl (mmol/l), Chloride.
*Significant difference vs. control, $p < 0.05$.

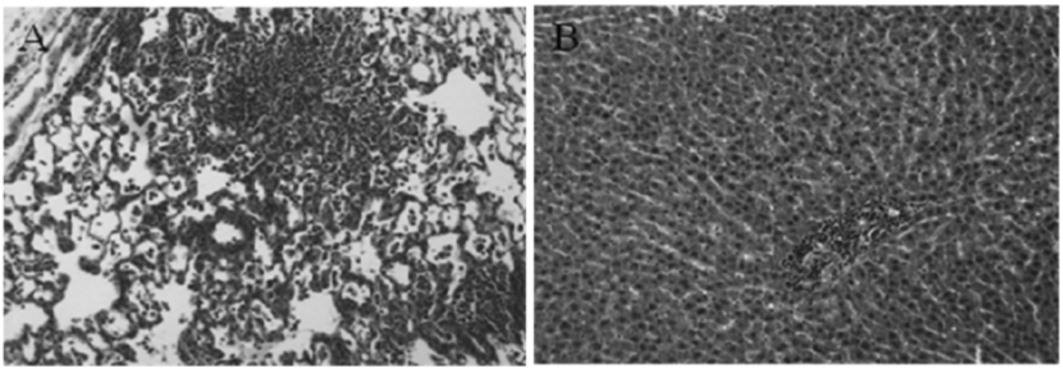

**Fig. 3.** Histopathological changes of male rats with exposed with cadmium selenide. (A) Control group, the rats appear focal inflammation in the lung. Hematoxylin & Eosin; Magnification: 50 ×. (B) 1,000 mg/kg group, the rats appear focal inflammation in the liver. Staining: Hematoxylin & Eosin; Magnification: 20 ×.

control group (Fig. 3).

## DISCUSSION

In this study, we performed to evaluate the toxicity of the 28-days repeated oral administration of CdSe using SD rats. The test substance was orally administrated daily at dose levels of 30, 300 and 1,000 mg/kg of body weight. Mortality and clinical observation were conducted, and body weight, water intakes and food consumption were recorded weekly.

Mortality, general observation, food consumption, water consumption, body weight changes, ophthalmoscopy, coagulation time and gross findings revealed no abnormalities in all treatment groups of both sexes.

In urinalysis, there were the significant increase of leukocyte frequency in male 300 and 1,000 mg/kg group and protein frequency in male 1,000 mg/kg group. In hematology and blood biochemistry analysis, there were the significant increase in IP in male 300 and 1,000 mg/kg group and the increase in CRE in female 30 mg/kg group. Significant increase in WBC and MO values in male 300 mg/kg group, and MO and RDW in male 1,000 mg/kg group, significant decrease in MCV in male 1,000 mg/kg group were observed. There were significant decrease in MCH in female 300 mg/kg group, MCV and MCH were significantly decreased in female 1,000 mg/kg group. But, these results were considered not toxicologically significant, because of these changes were within the limit of normal biological variation or not exhibit a dose-response relationship and not supported by pathological findings (Petterino *et al.*, 2006; Alemán *et al.*, 1996; Kang *et al.*, 1995). The decreased absolute weight of thymus in female 1,000 mg/kg group was negligible, not associated with the gross and histopathological examination.

Based on these results, it was suggested that twenty eight repeated oral dose of CdSe did not cause any toxic effect to SD rats at the dose level of 1,000 mg/kg under condition of this study. And the NOAEL of CdSe is considered to be over 1,000 mg/kg for both sexes.

## ACKNOWLEDGEMENT

This study was supported by Korean Ministry of Education, Science and Technology (Grant No. 2007-2005219)
